# Clinical comorbidity phenotypes in COPD and their biomarker signatures

**DOI:** 10.1183/23120541.00127-2026

**Published:** 2026-09-01

**Authors:** Line Egerod, Diana J. Leeming, Morten A. Karsdal, Julie C. Yates, Jannie M.B. Sand, Cecilie L. Bager

**Affiliations:** 1 Department of Biomedical Sciences, University of Copenhagen, Copenhagen, Denmark; 2 Nordic Bioscience, Herlev, Denmark; 3 GlaxoSmithKline R&D, Philadelphia, PA, USA

## Abstract

Comorbidities are common in COPD and clusters of co-occurring conditions (*i.e.* phenotypes) have been described [1, 2]. These phenotypes capture clinical profiles but do not explain the underlying biological endotypes. Biomarker studies can link phenotypes to endotypes, identify drivers of disease progression and suggest targets for intervention [3]. Systemic inflammation is often proposed as a shared driver of COPD and its comorbidities [1, 4] but direct evidence remains elusive.

## Shareable abstract

**Biomarkers of inflammation, coagulation and fibroblast activity differ across COPD comorbidity phenotypes, revealing biologically plausible signatures and suggesting potential endotypes that reflect subclinical disease processes** https://bit.ly/49t5nkW

*To the Editor:*

Comorbidities are common in COPD and clusters of co-occurring conditions (*i.e.* phenotypes) have been described [1, 2]. These phenotypes capture clinical profiles but do not explain the underlying biological endotypes. Biomarker studies can link phenotypes to endotypes, identify drivers of disease progression and suggest targets for intervention [3]. Systemic inflammation is often proposed as a shared driver of COPD and its comorbidities [1, 4] but direct evidence remains elusive. Systemic processes beyond inflammation may also contribute. Biomarkers of tissue remodelling and injury, including extracellular matrix (ECM) fragments, and markers of neutrophil activity and epithelial damage, have been proposed to be useful in COPD [5, 6], yet their relationship with comorbidities is largely unexplored.

Here, we investigated whether circulating biomarkers covering multiple systemic processes differ across previously identified comorbidity phenotypes of COPD [2] in the ECLIPSE cohort [7]. Our aim was to identify biological signatures linking these phenotypes to endotypes using established and emerging biomarkers.

The ECLIPSE [7] study enrolled 2164 COPD participants across multiple centres in 2005–2010. Participants were 40–75 years old, current or former smokers with ≥10 pack-years. Ethics approval was obtained for all centres.

Egerod *et al*. [2] identified five mutually exclusive comorbidity phenotypes in 2054 participants, which were used in this analysis: 1) no comorbidities (n=296); 2) musculoskeletal (n=562), mainly females with malnutrition; 3) mental (n=278), younger females with the greatest comorbidity burden; 4) circulatory (n=217), older males; and 5) metabolic (n=701), older males with excess body weight. Rules for assigning participants meeting multiple phenotypes were described previously [2].

Blood samples were analysed for 17 biomarkers reflecting inflammation (high-sensitivity C-reactive protein, interleukin (IL)-6, IL-8 and nordicVICM), coagulation (fibrinogen and nordicX-FIB), ECM degradation (nordicC3M and nordicC6M), fibroblast activity (Elecsys PRO-C3) and systemic stress (nordicPRO-C6), epithelial injury (CC-16, surfactant protein D, nordicC4M and nordicC4Ma3), and neutrophil activity (nordicELA-HNE, nordicELP-3 and nordicCPa9-HNE). Biomarker data were available for 1448–2029 (70.5–98.8%) participants. No imputation was performed as each biomarker was analysed separately excluding participants with missing data.

Biomarkers were logarithmically transformed and analysed using multivariable linear regression, with comorbidity phenotype membership (in *versus* out of phenotype) as the main predictor, adjusted for age, sex, body mass index (BMI), and smoking status. We report percentage differences derived from the regression coefficient (95% CI) and estimated marginal means back-transformed to the original scale to facilitate interpretation. A p-value <0.05 was considered significant. Analyses were conducted in R.

Figure 1 shows biomarker differences across the five comorbidity phenotypes. Participants without comorbidities had lower systemic stress and coagulation activity, reflected by reduced serum type VI collagen formation fragments (PRO-C6) (−5.1% (95% CI −9.7– −0.5%), p=0.03; 7.9 *versus* 8.3 ng·mL^−1^) and reduced plasma fibrinogen (−3.1% (95% CI −6.0– −0.2%, p=0.04; 381.1 *versus* 393.2 mg·dL^−1^). The musculoskeletal phenotype showed higher systemic inflammation but lower local inflammation, with serum IL-6 increased (+14.7% (95% CI +2.3– +27.2%), p=0.02; 2.6 *versus* 2.2 pg·mL^−1^) and serum IL-8 reduced (−12.8% (95% CI −25.2– −0.4%), p=0.04; 6.6 *versus* 7.4 pg·mL^−1^). The mental phenotype had lower IL-8 (−17.8% (95% CI −31.6– −4.0%), p=0.01; 6.2 *versus* 7.3 pg·mL^−1^). The circulatory phenotype showed higher IL-8 (+16.0% (95% CI +0.7– +31.2%), p=0.04; 8.3 *versus* 7.2 pg·mL^−1^) and lower neutrophil activity, reflected by reduced serum protease 3-mediated elastin degraded fragments (ELP-3) (−4.7% (95% CI −7.9– −1.5%), p=0.004; 6737.7 *versus* 7051.5 ng·mL^−1^). The metabolic phenotype had lower IL-6 (−16.1% (95% CI −27.6– −4.7%), p=0.006; 2.1 *versus* 2.4 pg·mL^−1^).)

**FIGURE 1:**
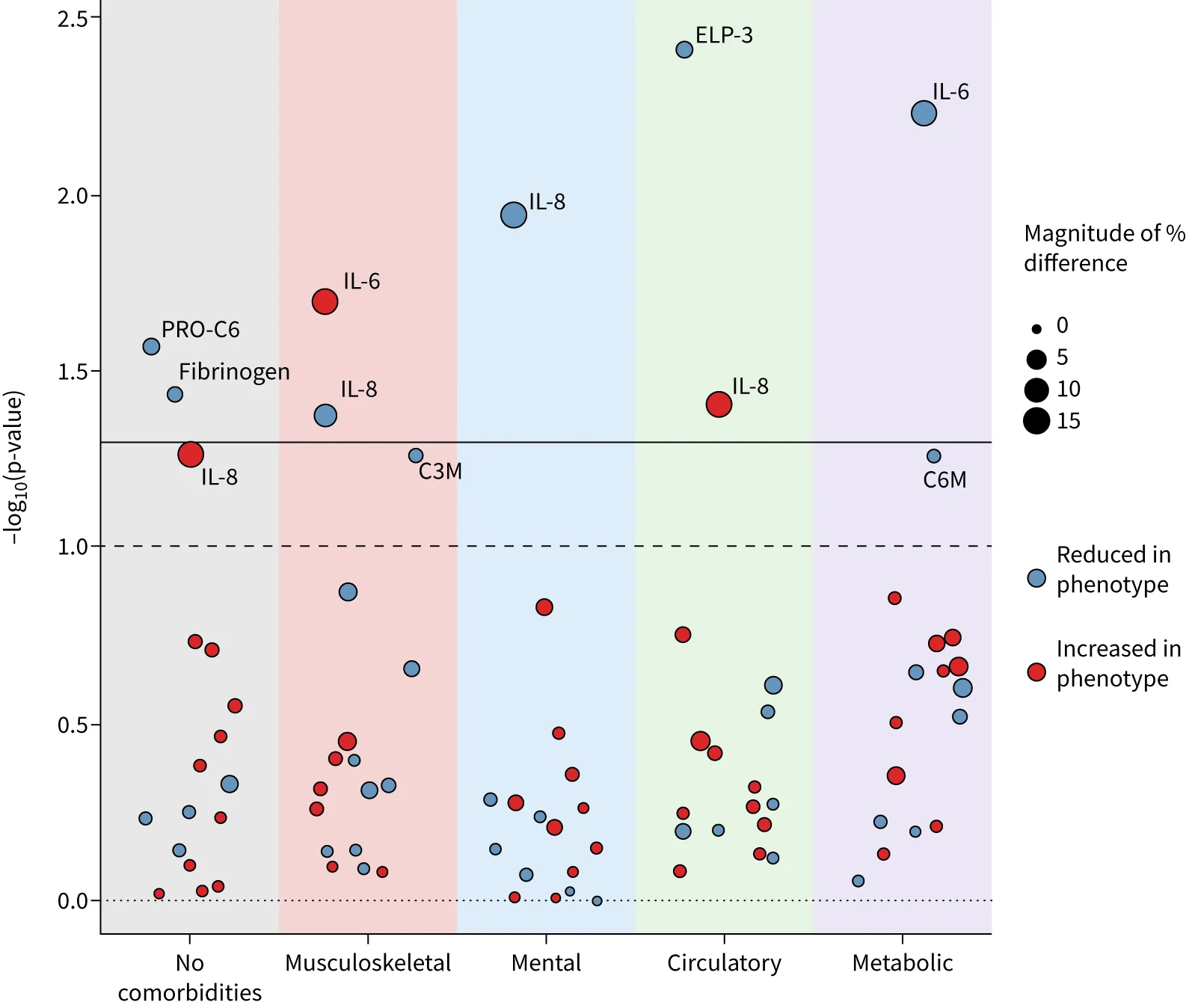
Associations between circulating systemic biomarkers and previously identified comorbidity phenotypes. Each dot represents a biomarker in one phenotype compared with all other phenotypes. Background shading separates the five phenotypes: no comorbidities (grey), musculoskeletal (red), mental (blue), circulatory (green) and metabolic (purple). The vertical axis reflects the statistical strength of the difference between in-phenotype and out-of-phenotype biomarker levels. Dot colour indicates direction (red: increased in the phenotype; blue: reduced in phenotype) and dot size indicates the magnitude of the difference. Solid and dashed horizontal lines indicate p=0.05 and p=0.1, respectively. Biomarkers with p<0.1 are labelled. ELP-3: protease 3-mediated elastin degraded fragments; IL: interleukin; PRO-C6: type VI collagen formation fragments.

Most biomarkers showed only modest variation across phenotypes, indicating limited specificity for individual comorbidities. Some findings align with previous studies, while others differ, highlighting the complexity of the underlying biology.

Fibrinogen and PRO-C6 were reduced in the no-comorbidity phenotype, representing the most clinically meaningful observation due to their marked differences compared with participants with comorbidities (12.1 mg·dL^−1^ and 0.4 ng·mL^−1^, respectively). Together, they may define a protective endotype marked by lower coagulation activity and systemic stress. Fibrinogen is an acute-phase protein and the only US Food and Drug Administration-qualified prognostic biomarker in COPD [8], with a proposed threshold of 350 mg·dL^−1^. Here, the unadjusted geometric mean was 386.9 mg·dL^−1^ [8], so most participants exceeded this cut-off. A single measurement may therefore be uninformative whereas monitoring longitudinally may be more useful, since rising levels over time could signal a transition between endotypes. A meta-analysis [9] found that a 1-g·L^−1^ increase in fibrinogen was associated with a hazard ratio of 2.03 for nonvascular mortality, corresponding to ∼9% higher risk per 12-mg·dL^−1^ increase.

PRO-C6 has been linked to mortality across chronic diseases [10]. One study reported healthy reference values of 6.9–8.7 ng·mL^−1^ (interquartile range (IQR)) [6] and levels observed here were within this range. This is consistent with the largely nonfibrotic pathology of COPD. PRO-C6 may instead qualify as a marker useful for detecting early comorbidity development, as levels have been shown to increase with comorbidity burden [11].

Combined, fibrinogen and PRO-C6 yielded an area under the curve of 0.57 (95% CI 0.53–0.61), indicating limited ability to discriminate between participants with and without comorbidities. Their utility may lie in monitoring changes within individuals over time rather than in cross-sectional prediction.

The neutrophil activity biomarker ELP-3 was lower in the circulatory phenotype, which includes participants with cardiovascular diseases, even though IL-8 was elevated. This is surprising, as IL-8 recruits monocytes and neutrophils during acute inflammation. The study [2] lacked data on comorbidity duration and severity, so these participants may represent later disease stages, meaning that neutrophils may have already degranulated and died, while IL-8 remains elevated as part of inflammatory feedback. Yet, despite an absolute difference of 313.8 ng·mL^−1^ between participants within and outside the phenotype, ELP-3 levels stayed above reported healthy reference values (IQR 3457–5160 ng·mL^−1^) [12]. This pattern suggests a common tissue-degradation endotype across COPD participants, reflecting ongoing elastin fragmentation consistent with disease pathology.

IL-8 or IL-6 showed phenotype-specific shifts across most phenotypes, suggesting inflammation-driven endotypes. IL-6 was elevated in the musculoskeletal phenotype, which included osteoporosis, muscle wasting and osteoarthritis. This supports a role for pro-inflammatory cytokines in these comorbidities. IL-8 was reduced in the same phenotype, suggesting a lower local inflammatory response. A similar pattern has been reported in chronic fatigue syndrome [13]. IL-8 was also reduced in the mental phenotype, defined by depression and anxiety, in line with reports on higher IL-8 levels being protective against depression [14]. Unexpectedly, IL-6 was lower in the metabolic phenotype, defined by obesity, hypertension and diabetes. Elevated IL-6 is often cited as a mediator of metabolic disease [15], largely through secretion from adipose tissue. In our analysis, IL-6 was adjusted for BMI. The lower levels therefore suggest that these participants were less systemically inflamed and may represent a healthier comorbidity phenotype.

However, absolute differences were small (<1.2 pg·mL^−1^). Although biologically plausible, their clinical impact may be limited. IL-6 and IL-8 act in multiple pathways, and are unlikely to define distinct endotypes on their own. The modest shifts observed here may reflect overlapping mechanisms rather than separate inflammatory processes.

This study has limitations. It is observational and comorbidities were recorded before enrolment, limiting prognostic interpretation. Only biomarkers with data available to us were analysed, potentially excluding relevant biological pathways. Analyses were restricted to a single cohort without external validation. Further research is needed to clarify mechanisms and confirm biomarker–endotype associations.

In summary, circulating biomarkers showed modest differences across COPD comorbidity phenotypes in ECLIPSE, with some displaying phenotype-specific associations. These patterns are biologically plausible and may reflect underlying endotypes. Most absolute differences were small and unlikely to guide clinical monitoring. In contrast, low fibrinogen and PRO-C6 identified participants without comorbidities. Rising levels of these markers over time may indicate emerging comorbidity and worse prognosis, suggesting their potential for monitoring disease progression.
